# Impacto del Modelo Trinomio Asistencial en la accesibilidad de la Atención Primaria: estudio cuasiexperimental antes-después

**DOI:** 10.1016/j.aprim.2026.103522

**Published:** 2026-05-27

**Authors:** Fátima Cañas Tornero, Manuel Ignacio Monge García, Lourdes González Soria, Andrés Rabadán Asencio

**Affiliations:** a Área de Gestión Sanitaria Jerez, Hospital Universitario Jerez de la Frontera, Servicio Andaluz de Salud Costa Noroeste y Sierra de Cádiz, Andalucía, España; b Unidad de Cuidados Intensivos, Hospital Universitario de Puerto Real, Puerto Real, España; c Área de Gestión Sanitaria Jerez, Centro de Salud La Serrana, Servicio Andaluz de Salud, Costa Noroeste y Sierra de Cádiz, Andalucía, España; d Escuela Andaluza de Salud Pública, Granada, España

**Keywords:** Atención Primaria de Salud, Accesibilidad a los Servicios de Salud, Innovación organizacional, Listas de espera, Gestión sanitaria, Primary Health Care, Health Services Accessibility, Organizational Innovation, Waiting Lists, Health Care Management

## Abstract

**Objetivo:**

Evaluar el impacto del Modelo Trinomio Asistencial (MTA) sobre la accesibilidad en Atención Primaria mediante el análisis del Tiempo Medio de Respuesta (TMR).

**Diseño:**

Estudio cuasiexperimental multicéntrico, retrospectivo, con diseño antes-después.

**Emplazamiento:**

Tres centros de salud de la Zona Básica de Salud (ZBS) de Jerez, pertenecientes al Área de Gestión Sanitaria (AGS) Jerez, Costa Noroeste y Sierra de Cádiz (Andalucía, España).

**Participantes:**

Se incluyeron 30 profesionales médicos con continuidad asistencial durante todo el periodo de estudio. Se analizaron los registros correspondientes a los dos meses previos y los dos meses posteriores a la implementación del modelo, excluyendo la fase de implantación y los meses estivales.

**Intervenciones:**

Implementación del MTA, basado en la integración funcional de medicina de familia, enfermería y la Unidad de Atención a la Ciudadanía (UAC) para la reorganización de la demanda asistencial.

**Mediciones principales:**

TMR, definido como el número de días hasta la obtención de cita médica programada.

**Resultados:**

La mediana del TMR se redujo de 8,93 días (preintervención) a 4,61 días (posintervención), lo que supuso una reducción relativa mediana del 39,5% (rango intercuartil [RIC]: 31,1–55,3; p < 0,0001). La mejora fue consistente en 28 de los 30 profesionales. El análisis multivariable no mostró asociación significativa entre la variación del TMR y las características profesionales ni la carga asistencial.

**Conclusiones:**

El MTA se asocia a una mejora significativa y clínicamente relevante de la accesibilidad en Atención Primaria, atribuible al rediseño organizativo del equipo y no a factores individuales de los profesionales.

## Abstract (en)

**Objective:**

To assess the impact of the Healthcare Triad Model (HTM) on Primary Care accessibility by analyzing changes in Average Response Time (ART).

**Design:**

Multicenter, retrospective, quasi-experimental before-and-after study.

**Setting:**

Three primary care centers within the Jerez, Costa Noroeste and Sierra de Cádiz Health Management Area (Andalusia, Spain).

**Participants:**

Thirty physicians with continuous clinical activity throughout the study period. Data from the two months before and two months after implementation were analyzed, excluding the rollout phase and summer months.

**Interventions:**

Implementation of the Healthcare Triad Model, which integrates family physicians, nurses, and citizen care units to reorganize care demand management.

**Main outcome measure:**

Average Response Time (ART), defined as the number of days until a scheduled medical appointment.

**Results:**

Median ART decreased from 8.93 to 4.61 days, representing a median relative reduction of 39.5% (interquartile range [IQR]: 31.1–55.3; p < 0.0001). Improvement was observed in 28 of 30 physicians. Multivariable analysis showed no significant associations between ART reduction and individual professional or workload characteristics.

**Conclusions:**

The Healthcare Triad Model significantly improves Primary Care accessibility through organizational redesign, independently of individual professional factors.

## Introducción

La Atención Primaria constituye el eje vertebrador de los sistemas sanitarios públicos. Uno de sus atributos esenciales es la accesibilidad^1^, ^2^, entendida como la capacidad del sistema para ofrecer una respuesta oportuna, adecuada y centrada en las necesidades de las personas. En los últimos años, el incremento sostenido de la demanda asistencial, la escasez de profesionales y la creciente burocratización han comprometido de forma significativa este atributo, generando demoras, sobrecarga profesional y una pérdida progresiva de calidad percibida por la ciudadanía.

La transformación de la Atención Primaria requiere avanzar hacia modelos organizativos basados en la atención centrada en la persona, la integración funcional de los equipos y el uso eficiente de los recursos disponibles. La evidencia señala que los sistemas con una Atención Primaria fuerte, accesible y resolutiva obtienen mejores resultados en salud, mayor equidad y sostenibilidad^3^. En este contexto, la innovación organizativa no implica necesariamente la incorporación de nuevos recursos, sino la reorganización de los existentes mediante estructuras más flexibles, colaborativas y orientadas a competencias^4^, ^5^, ^6^.

El Modelo Trinomio Asistencial (MTA) se fundamenta en estos principios y propone una reorganización del funcionamiento interno de los centros de salud a partir de la integración corresponsable de tres perfiles profesionales clave —medicina de familia, enfermería y Unidad de Atención a la Ciudadanía (UAC)—, junto con la participación activa de la ciudadanía. El MTA se apoya en la gestión estructurada de la demanda desde el primer contacto, la redistribución de tareas según competencias, la desburocratización de la consulta médica y la resolución en acto único como elementos nucleares para mejorar la accesibilidad. Desde una perspectiva funcional, el modelo introduce cambios en los circuitos asistenciales cotidianos, reorganizando la entrada al sistema, redefiniendo las agendas profesionales y favoreciendo la coordinación interna del equipo^7^, ^8^, ^9^, ^10^.

El desarrollo del MTA fue precedido por un proceso de pilotaje llevado a cabo entre 2021 y 2023 en el Centro de Salud de La Serrana (Jerez de la Frontera), donde la participación activa del equipo profesional permitió el diseño, ajuste y maduración del modelo en condiciones reales de práctica asistencial. Tras los resultados favorables obtenidos, la Dirección Gerencia del Área de Gestión Sanitaria (AGS) Jerez, Costa Noroeste y Sierra de Cádiz impulsó la implantación progresiva del modelo en otros centros del área durante 2024. En la actualidad, el MTA se encuentra implementado en todos los centros de salud y consultorios locales del AGS, constituyendo el marco organizativo de referencia para la gestión de la demanda asistencial.

El objetivo del presente estudio fue evaluar el impacto del MTA sobre el Tiempo Medio de Respuesta (TMR) en Atención Primaria, como indicador cuantitativo de accesibilidad.

## Material y métodos

### Diseño y ámbito del estudio

**Figura 1:**
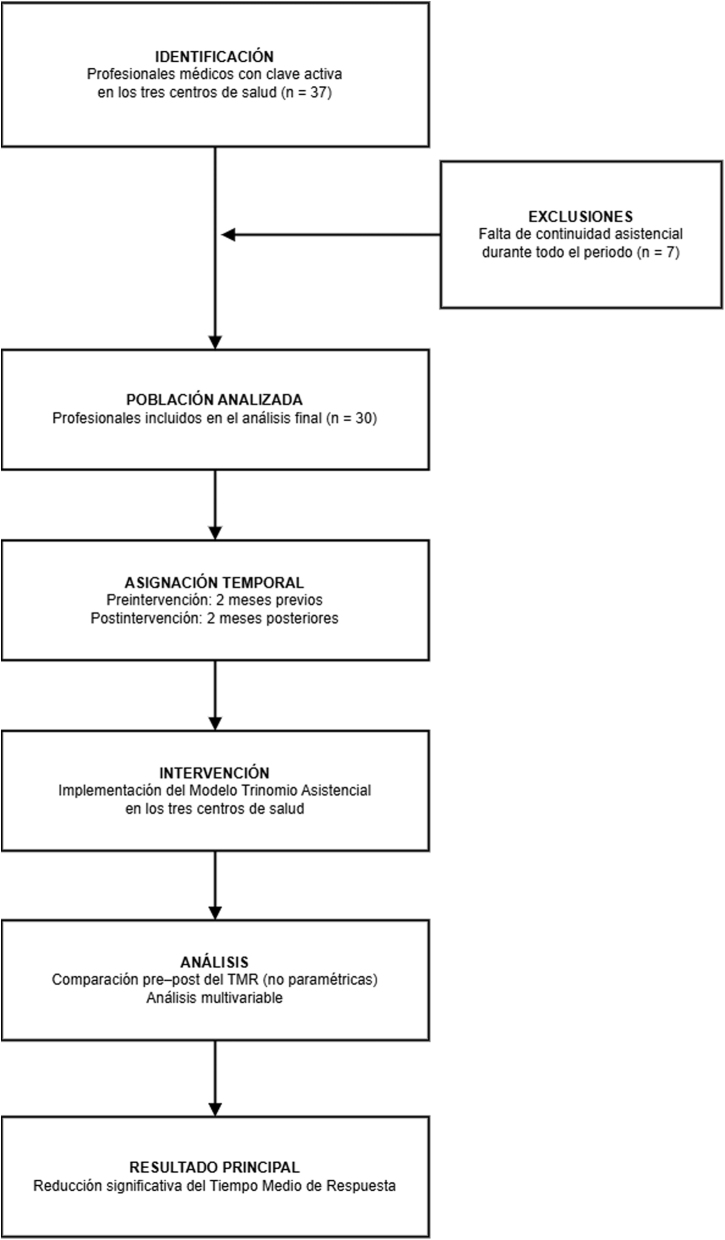
Estudio cuasiexperimental antes-después, multicéntrico, sin grupo control externo, con evaluación del impacto organizativo sobre la accesibilidad en Atención Primaria.

Se realizó un estudio cuasiexperimental multicéntrico, retrospectivo, con diseño antes-después (fig. 1), en tres centros de salud de la Zona Básica de Salud (ZBS) de Jerez, pertenecientes al AGS Jerez, Costa Noroeste y Sierra de Cádiz (Andalucía, España): Centro de Salud Jerez Sur, Centro de Salud La Milagrosa y Centro de Salud Jerez Centro. Los tres centros fueron seleccionados intencionadamente por sus diferencias organizativas y poblacionales, a pesar de estar integrados en una misma área sanitaria.

### Población de estudio

La población estuvo constituida por profesionales médicos con clave activa y continuidad asistencial durante todo el periodo de estudio. Se excluyeron aquellos sin continuidad asistencial durante todo el periodo analizado (ausencias prolongadas, cambios de destino o modificaciones estructurales de cupo). De los 37 profesionales con clave médica activa en los tres centros, se incluyeron 30 en el análisis final.

### Intervención

La intervención evaluada consistió en la implementación del MTA como estrategia de reorganización funcional^1^, ^11^. El MTA se articula como una célula asistencial estable formada por medicina de familia, enfermería y la UAC, que actúan de manera coordinada y corresponsable. El modelo reorganiza el acceso al sistema desde el primer contacto del paciente mediante un sistema de turnos que clasifica la demanda en consultas no demorables, gestiones administrativas o solicitud de cita programada. La UAC realiza una primera valoración y canaliza la demanda; la enfermería asume un rol central en la consulta de acogida, resolviendo directamente las demandas dentro de su ámbito competencial y derivando al médico solo los casos que requieren atención médica específica. El profesional médico concentra su actividad en problemas de mayor complejidad, con agendas rediseñadas que incorporan tramos flexibles y consultas no demorables distribuidas equitativamente^7^, ^12^, ^13^, ^14^. Como componente transversal, el modelo integra la participación activa de la ciudadanía a través de las Comisiones de Participación Ciudadana^2^, ^15^, ^16^. La descripción detallada de las líneas estratégicas, los circuitos operativos y la estructura de las agendas profesionales se recoge en el material suplementario (Anexos 1–4).

### Variables y fuentes de información

La variable principal fue el TMR, definido como el número de días hasta la obtención de cita médica programada. Se construyó una base de datos a partir del registro diario del TMR de cada clave médica, obtenido de la aplicación corporativa MTI Citas del Servicio Andaluz de Salud. Se seleccionaron los dos meses previos a la implementación del modelo (periodo preintervención) y los dos meses posteriores a su consolidación (periodo posintervención), excluyendo la fase de implantación y los meses estivales para evitar sesgos estacionales. Como variables secundarias se recogieron las características profesionales (edad, sexo, antigüedad en el centro) y los indicadores de carga asistencial: tarjetas individuales sanitarias (TISS) y tarjetas ajustadas por edad y sexo (TAES), obtenidos de las aplicaciones GERHONTE e INFOWEB, respectivamente.

### Análisis estadístico

Las variables cuantitativas se describieron como mediana y rango intercuartil (rango intercuartil [RIC], percentiles 25–75) dado que no seguían una distribución normal (prueba de Shapiro-Wilk). La comparación del TMR antes y después de la intervención se realizó mediante la prueba de Wilcoxon para muestras pareadas. La comparación de la variación porcentual del TMR entre los tres centros se analizó mediante la prueba de Kruskal-Wallis, seguida del análisis *post-hoc* de Conover para identificar diferencias específicas entre pares de centros.

La influencia de las variables profesionales y de carga asistencial sobre la reducción del TMR se evaluó mediante un modelo de regresión lineal múltiple por pasos (*stepwise*), considerando como variable dependiente la variación porcentual del TMR y como variables independientes la edad, la antigüedad, el sexo, el número de TISS y el número de TAES. Se consideró un valor de p < 0,05 como estadísticamente significativo. El análisis se realizó con MedCalc® versión 23.2.2 (MedCalc Software Ltd, Ostend, Bélgica).

### Consideraciones éticas

El estudio se realizó con datos agregados procedentes de sistemas de información corporativos del Servicio Andaluz de Salud, sin acceso a datos clínicos individuales de pacientes ni a muestras biológicas. La intervención evaluada correspondió a una decisión organizativa institucional, no a una intervención con fines de investigación. De acuerdo con la normativa vigente, no se requirió aprobación por parte de un Comité de Ética de la Investigación.

## Resultados

Se incluyeron 30 profesionales médicos (24 mujeres [80%]; edad media: 51,3 años) pertenecientes a tres centros de salud: Jerez Sur (n = 14; 19.031 TISS), La Milagrosa (n = 9; 13.561 TISS) y Jerez Centro (n = 7; 11.649 TISS). Los datos descriptivos de la población se detallan en la tabla 1.

**Tabla 1:** Características de los profesionales incluidos en el estudio, por centros

	C.S. Jerez Sur (n = 14)	C.S. La Milagrosa (n = 9)	C.S. Jerez Centro (n = 7)
Edad, años	54,3	51,0	45,6
Mujeres, n (%)	11 (78,6)	8 (88,9)	5 (71,4)
Antigüedad, días, mediana (RIC)	2.218 (945–2.891)	1.429 (1.132–1.430)	519 (484–1.131)
TISS, mediana (RIC)	1.399 (1.372–1.444)	1.558 (1.522–1.606)*	1.297 (1.200–1.462)
TAES, mediana (RIC)	1.919 (1.849–2.002)	2.003 (1.983–2.154)	1.947 (1.746–2.193)
Claves médicas activas	17	11	10

TISS: Tarjetas Individuales Sanitarias; TAES: tarjetas ajustadas por edad y sexo; RIC: rango intercuartil.

* Diferencias significativas entre centros (Kruskal-Wallis, p = 0,004).

Tras la implementación del MTA, la mediana del TMR global descendió de 8,93 días (RIC: 6,34–10,00) en el periodo preintervención a 4,61 días (RIC: 4,04–5,69) en el posintervención, lo que supuso una reducción relativa mediana del 39,5% (RIC: 31,1–55,3; prueba de Wilcoxon, p < 0,0001). Esta mejora se produjo sin incremento de recursos humanos ni modificaciones estructurales relevantes. A nivel individual, 28 de los 30 profesionales (93,3%) presentaron una reducción del TMR tras la intervención (Anexo 5).

El análisis por centros mostró diferencias en la magnitud del efecto. En el centro de salud Jerez Sur la mediana del TMR descendió de 9,55 días (RIC: 8,41–10,36) a 4,06 días (RIC: 3,84–4,29), con una reducción relativa del 56,1% (RIC: 51,2–60,3; p = 0,0001). En el C.S. La Milagrosa descendió de 9,29 días (RIC: 8,87–10,00) a 5,83 días (RIC: 5,53–6,21), con una reducción del 38,8% (RIC: 35,2–39,9; p = 0,004). En el centro de salud. Jerez Centro, que partía de valores basales más favorables, la mediana descendió de 5,73 días (RIC: 5,35–6,21) a 5,51 días (RIC: 4,66–5,91), sin alcanzar significación estadística (p = 0,078), probablemente con relación al menor tamaño muestral (n = 7) y el menor margen de mejora. Estos resultados se resumen en la tabla 2.

**Tabla 2:** Tiempo Medio de Respuesta antes y después de la implementación del Modelo Trinomio Asistencial, global y por centros

Centro (n)	TMR pre Mediana (RIC)	TMR post Mediana (RIC)	Reducción % Mediana (RIC)	p	Mejoraron n (%)
Global (30)	8,93 (6,34–10,00)	4,61 (4,04–5,69)	39,5 (31,1–55,3)	< 0,0001	28 (93,3)
Jerez Sur (14)	9,55 (8,41–10,36)	4,06 (3,84–4,29)	56,1 (51,2–60,3)	0,0001	14 (100)
La Milagrosa (9)	9,29 (8,87–10,00)	5,83 (5,53–6,21)	38,8 (35,2–39,9)	0,004	9 (100)
Jerez Centro (7)	5,73 (5,35–6,21)	5,51 (4,66–5,91)	—	0,078	5 (71,4)

TMR: Tiempo Medio de Respuesta (días); RIC: rango intercuartil (percentiles 25–75).

Prueba de Wilcoxon para muestras pareadas. En Jerez Centro no se indica reducción porcentual al no alcanzar significación estadística.

**Figura 2:**
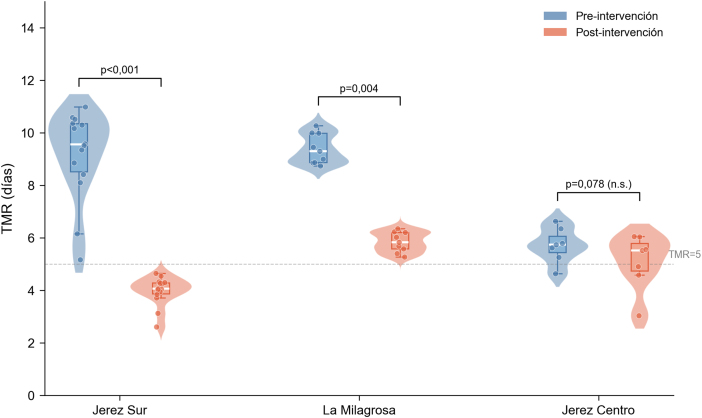
Distribución del Tiempo Medio de Respuesta (TMR) antes y después de la implementación del Modelo Trinomio Asistencial, por centro de salud. Diagrama *raincloud* que combina gráfico de violín, *boxplot* y datos individuales.

La figura 2 muestra la distribución del TMR pre y posintervención para cada centro de salud.

La comparación de la variación del TMR entre centros mediante la prueba de Kruskal-Wallis confirmó diferencias estadísticamente significativas (H = 23,36; p < 0,001). El análisis *post-hoc* de Conover mostró diferencias significativas entre los tres pares de centros, lo que sugiere que la magnitud del efecto se modula por el punto de partida organizativo de cada dispositivo.

En el análisis de regresión múltiple por pasos no se retuvo ninguna variable en el modelo, indicando que ni la edad, ni el sexo, ni la antigüedad, ni los indicadores de carga asistencial (TISS, TAES) se asociaron de forma significativa con la variación del TMR. La prueba de Shapiro-Wilk sobre los residuos del modelo mostró una desviación marginal de la normalidad (W = 0,930; p = 0,048), lo que debe considerarse al interpretar estos resultados.

## Discusión

Este estudio muestra que la implementación del MTA se asoció a una reducción significativa del TMR, con una magnitud cercana al 40% en el análisis global. Este resultado es clínicamente relevante en un contexto caracterizado por la sobrecarga asistencial y las limitaciones estructurales de la Atención Primaria, donde las demoras en la atención representan una de las principales fuentes de insatisfacción de la ciudadanía y de erosión del vínculo profesional-paciente.

Los hallazgos son concordantes con experiencias previas que han demostrado el impacto positivo de las intervenciones organizativas sobre la accesibilidad. En el ámbito nacional, el Proyecto Tarragona documentó mejoras en la capacidad resolutiva y en los tiempos de atención mediante la reorganización de circuitos asistenciales y la potenciación del trabajo en equipo^17^. Asimismo, Coll Benejam et al.^14^, describieron los principales cambios organizativos adoptados en Atención Primaria durante la pandemia de COVID-19, incluyendo la reorganización de circuitos asistenciales y dinámicas de trabajo para mantener la accesibilidad. Casajuana^6^ ha subrayado que la desburocratización y el principio de prudencia en la consulta médica constituyen estrategias eficaces para liberar tiempo clínico. Estos resultados coinciden con los observados en el presente estudio y respaldan la plausibilidad del efecto atribuido al MTA.

En el ámbito internacional, el modelo *Primary Care Home* del National Health Service británico ha mostrado que la integración funcional de perfiles profesionales y el rediseño de flujos de acceso mejoran la eficiencia y la experiencia del paciente^18^. La evidencia internacional confirma que el liderazgo clínico local y la flexibilidad organizativa son factores determinantes del éxito de estas intervenciones^3^. El MTA comparte con estos modelos el enfoque integrador y resolutivo, pero se diferencia en dos aspectos: la incorporación explícita del personal administrativo como miembro activo del equipo asistencial y su escalamiento a la totalidad de centros de un AGS, superando el carácter local o experimental de experiencias previas.

La heterogeneidad observada entre centros merece una consideración específica. El C.S. Jerez Sur, con los valores basales más desfavorables, obtuvo la mayor reducción (56%), mientras que el C.S. Jerez Centro, con un TMR basal inferior, mostró un efecto más discreto y no significativo. Este patrón, consistente con lo descrito en la literatura sobre modelos de cambio organizativo^19^, ^20^, sugiere que el margen de mejora es proporcional al grado de desequilibrio previo y que el impacto del modelo depende del contexto organizativo de cada centro.

La ausencia de asociación entre la reducción del TMR y las características individuales de los profesionales refuerza la hipótesis de que el impacto del MTA depende del rediseño estructural y no del rendimiento individual. Este hallazgo tiene implicaciones prácticas relevantes: señala que las estrategias de mejora de la accesibilidad deben orientarse hacia la reorganización de los procesos asistenciales más que a las intervenciones centradas en el profesional aislado.

El estudio presenta limitaciones. El diseño cuasiexperimental antes-después, sin grupo control externo, limita la inferencia causal y no permite excluir completamente la influencia de factores temporales concomitantes. El tamaño muestral, especialmente en el C.S. Jerez Centro (n = 7), limita la potencia estadística para detectar diferencias significativas a nivel de centro. La desviación marginal de la normalidad de los residuos de la regresión (p = 0,048) sugiere cautela en la interpretación del análisis multivariable. No obstante, la utilización de periodos comparables, la exclusión de fases de implantación y meses estivales, la consistencia del efecto en 28 de 30 profesionales y la concordancia de los resultados entre centros refuerzan la validez interna de los hallazgos. Asimismo, la exclusión de profesionales sin continuidad asistencial, aunque metodológicamente justificada, pudo restringir la representatividad de la muestra.

Futuros estudios deberían evaluar la sostenibilidad del efecto a medio y largo plazo, incorporar indicadores complementarios como la satisfacción de usuarios y profesionales, la continuidad asistencial o los resultados en salud, y explorar mediante metodología cualitativa los factores facilitadores y las barreras percibidas en la implementación. Adicionalmente, sería de interés ampliar el análisis a muestras más heterogéneas de profesionales que incluyan distintos grados de estabilidad asistencial.

En conclusión, el MTA constituye una estrategia organizativa eficaz, viable y replicable para mejorar la accesibilidad en Atención Primaria, reduciendo significativamente los tiempos de respuesta sin incremento de recursos.

**Puntos clave**

**Lo conocido sobre el tema**

• La accesibilidad es uno de los principales retos actuales de la Atención Primaria, con tiempos de respuesta prolongados que generan insatisfacción y sobrecarga profesional.
• La reorganización de la demanda y la redistribución de tareas según competencias se han propuesto como estrategias de mejora, pero su evaluación cuantitativa en condiciones reales es limitada.
• La mayoría de las experiencias organizativas previas tienen carácter local o experimental, sin evidencia de escalamiento a nivel de área sanitaria.

**¿Qué aporta este estudio?**

• Aporta evidencia cuantitativa del impacto de un modelo organizativo innovador sobre la accesibilidad, con una reducción del 39,5% del TMR.
• Demuestra que la mejora es atribuible al rediseño organizativo y no a factores individuales de los profesionales.
• Describe un modelo ya implementado a escala de toda un AGS, viable y replicable sin incremento de recursos.

## Autoría

Fátima Cañas Tornero: Conceptualización, Metodología, Investigación, Administración del proyecto, Redacción del borrador original. Manuel Ignacio Monge García: Metodología, Análisis formal, Visualización, Redacción: revisión y edición. Lourdes González Soria: Investigación, Recursos, Curación de datos. Andrés Rabadán Asencio: Metodología, Supervisión, Redacción: revisión y edición.

## Financiación

Este estudio no ha recibido financiación específica.

## Consideraciones éticas

El estudio se realizó con datos agregados procedentes de sistemas de información corporativos del Servicio Andaluz de Salud, sin acceso a datos clínicos individuales de pacientes ni a muestras biológicas. La intervención evaluada correspondió a una decisión organizativa institucional, no a una intervención con fines de investigación. De acuerdo con la normativa vigente, no se requirió aprobación por parte de un Comité de Ética de la Investigación.

## Declaración sobre el uso de inteligencia artificial generativa

Durante la preparación de este manuscrito se utilizó una herramienta de inteligencia artificial generativa como apoyo en tareas de revisión, edición y verificación de datos. Los autores revisaron y editaron el contenido generado y asumen plena responsabilidad sobre el contenido final del artículo.

## Conflicto de intereses

Los autores declaran no tener ningún conflicto de intereses.
