# Supplementary material for: Impact of the Healthcare Triad Model on Primary Care accessibility: a quasi-experimental before-and-after study
Source: Aten Primaria. 2026 May 27;58(8):103522. [Article in Spanish] doi: 10.1016/j.aprim.2026.103522 (PMC13235447; doi:10.1016/j.aprim.2026.103522)
Supplement: Supplementary file 1 [file mmc1.doc]

**MATERIAL SUPLEMENTARIO**

Artículo: Impacto del Modelo Trinomio Asistencial en la accesibilidad de la Atención Primaria

Autora: Dra. Fátima Cañas Tornero

**Anexo 1. Líneas estratégicas del Modelo Trinomio Asistencial**

El Modelo Trinomio Asistencial se fundamenta en una reorganización funcional de la Atención Primaria orientada a mejorar la accesibilidad, la eficiencia y la adecuación de la respuesta asistencial.

Línea Estratégica 1: Integración operativa de los profesionales de la Unidad de Atención a la Ciudadanía en los equipos asistenciales

Esta línea estratégica es, sin duda, la más innovadora del modelo. Aunque la figura del profesional de la UAC ya estaba presente en los centros, aquí se le da un rol totalmente distinto: deja de ser un apoyo administrativo y pasa a ser una pieza clave en la organización de la demanda y en el funcionamiento del equipo.

No se trata solo de que la UAC forme parte del equipo, sino de que tenga herramientas reales, espacio propio y capacidad para decidir, como ya tienen otros perfiles. Eso es lo que hace que este modelo funcione: que, desde la entrada, la UAC pueda canalizar al paciente según su necesidad, resolver directamente lo que esté en su mano y derivar al profesional más adecuado si hace falta.

Para lograr esto, cada binomio médico-enfermero debe estar acompañado por un profesional de la UAC, creando así el Trinomio Asistencial. Además, se deben ajustar circuitos, rediseñar agendas y establecer una comunicación diaria, fluida y efectiva entre los tres perfiles. Esta integración no es solo formal, sino también operativa: el profesional de la UAC comprende los flujos del centro, coordina con su profesional médico y de enfermería de referencia, y facilita que el paciente no tenga que perder tiempo repitiendo su problema clínico en distintos puntos de atención.

Este cambio exige también una evolución en la forma de trabajar del equipo. Se deja atrás el modelo individual para dar paso a una dinámica basada en el reparto de responsabilidades**,** la colaboración en las tareas y un seguimiento conjunto de los casos. Lo que empieza en la puerta, se resuelve de forma compartida, y eso debe repercutir en una menor carga asistencial y una experiencia más satisfactoria para el paciente.

En definitiva, este equipo ampliado y bien engranado no solo reorganiza el trabajo: mejora los resultados. El Trinomio Asistencial es una forma más ordenada, más resolutiva y, sobre todo, más humana de entender la Atención Primaria. Centrada en la persona, ajustada a la realidad del centro y diseñada para ofrecer respuestas reales a las necesidades asistenciales.

Línea Estratégica 2: Gestión eficiente de la demanda asistencial

Esta línea estratégica constituye uno de los pilares del modelo. Su objetivo es claro: ordenar el acceso a los recursos disponibles, garantizando que cada demanda sea dirigida desde el primer momento al profesional más adecuado, optimizando los tiempos de atención y evitando saturaciones innecesarias.

1. *Automatización de la entrada y categorización por necesidad*. El uso de un *turnómetro* como punto de entrada permite al usuario clasificar su demanda de forma directa y autónoma desde que accede al centro. Esta automatización, lejos de sustituir al profesional, complementa su intervención permitiendo que la UAC valide y canalice cada caso según su naturaleza y complejidad. Se gana agilidad, se reducen errores en la asignación de citas y se reduce la presión sobre los mostradores.
2. *Implementación efectiva de agendas administrativas gestionadas por UAC*. Un avance clave será la consolidación de agendas específicas para trámites administrativos, que serán gestionadas directamente por el personal de la UAC. Estas agendas estarán disponibles tanto en el propio centro como a través de canales externos como Salud Responde o ClicSalud+. A través de ellas, el usuario podrá reservar de forma autónoma una cita para trámites como:
   - Cambios de domicilio o datos personales.
   - Solicitud de médico o centro.
   - Altas en el Sistema Sanitario.
   - Solicitud o recogida de documentación
   - Otras gestiones

Estas citas son presenciales y están organizadas en horarios y tramos diarios, lo que permite al equipo de la UAC estructurar su trabajo de manera eficiente, reducir tiempos de espera y agilizar la resolución de trámites. Este enfoque contribuye a ordenar el flujo de atención, evitar interrupciones en la actividad clínica y desburocratizar las consultas médicas. Asimismo, fortalece el papel de la UAC como primer punto resolutivo, fomenta su autonomía y empodera al usuario al ofrecerle mayor control sobre la gestión de su demanda, sin necesidad de depender exclusivamente del mostrador físico.

1. *Consulta de acogida. L*a consulta de acogida de enfermería constituye también otra pieza clave de este modelo. Permite que el usuario con una demanda clínica sea atendido de forma estructurada, inmediata y resolutiva desde el primer contacto, sin tener que esperar una consulta médica que, en muchos casos, no será necesaria. Para llevar a cabo este punto, el profesional de enfermería deberá llevar a cabo:

- Una valoración inicial estructurada
- Una resolución directa, si está dentro de su ámbito competencial
- Una derivación al médico, si la demanda requiere atención médica

Gracias a esta consulta, se potenciará el trabajo colaborativo con medicina, se evitará el paso innecesario por varios puntos del sistema y se favorecerá la resolución en acto único. La consulta de acogida se convertirá en un filtro clínico activo, que descongestionará el sistema, mejorará los tiempos de respuesta y permitirá que el paciente se sienta atendido desde el primer momento, no solo citado.

Línea Estratégica 3: Desburocratización

Una de las estrategias más revolucionarias del Modelo Trinomio Asistencial es la clara apuesta por reducir la carga burocrática en las consultas médicas. Este enfoque busca liberar tiempo para que los profesionales puedan centrarse exclusivamente en aspectos clínicos y se ha implementado a través de dos vías complementarias:

1. *Derivación de trámites administrativos fuera de la consulta médica*. Se implementa un circuito organizativo que permite redirigir al ámbito administrativo aquellos procedimientos que, durante años, se han realizado impropiamente desde la consulta médica. Entre las gestiones que ahora se tramitan directamente desde la UAC o por personal administrativo destacan:

- Justificantes de asistencia
- Solicitudes de transporte sanitario
- Fe de vida
- Informes no clínicamente justificados
- Faltas escolares justificadas por padres/tutores
- Solicitudes de partes o documentos sin relevancia médica directa

Este cambio cuenta con el respaldo normativo emitido por el SAS, consolidado a través de una instrucción precisa de la Dirección General de Asistencia Sanitaria: “El personal médico no deberá asumir trámites burocráticos que no correspondan a su competencia” (Direccion General de Asistencia Sanitaria y Resultados en Salud, 2023). Con esta medida se busca proteger el tiempo destinado a las actividades clínicas, profesionalizar los circuitos administrativos y liberar al personal médico de tareas impropias, mejorando así tanto la calidad como la eficiencia de la atención sanitaria.

1. *Implantación efectiva de consultas médicas en acto único*. Otro gran avance del modelo representa la incorporación real y funcional de la consulta en acto único. Este enfoque permite resolver el mayor número posible de gestiones clínicas en un solo contacto asistencial, evitando citas repetidas innecesarias, duplicidades y trámites posteriores superfluos. Entre las actuaciones que se integran de forma estructurada en este modelo destacan:

- *Peticiones de análisis de sangre*. Cuando la solicitud se realiza durante una consulta presencial, el médico entrega la petición al paciente, quien puede acudir directamente al mostrador para elegir la fecha que más le convenga. En caso de consulta telefónica, se indica al paciente que visite la UAC en el momento que prefiera; allí le imprimirán la petición y le asignarán la cita al instante.
- *Tareas específicas de enfermería generadas desde la consulta médica*. Estas son programadas directamente desde la consulta, utilizando agendas específicas previamente consensuadas con el enfermero o enfermera del equipo trinomio. Este método asegura una continuidad y coordinación efectiva en el proceso asistencial.
- *Teleconsultas*. Tras una consulta con el especialista que genere un diagnóstico y tratamiento, el médico de familia informa al paciente mediante una llamada telefónica. El informe se registra en la historia clínica y, si el paciente solicita una copia impresa, esta se envía a su domicilio por correo postal a través de la UAC, evitando desplazamientos innecesarios y facilitando el acceso a la información.
- *Derivaciones a especialista*. Estas se gestionan digitalmente desde la consulta médica, sin necesidad de imprimir en papel ni de que el paciente pase por el mostrador. El personal de la UAC contacta telefónicamente con el paciente para informarle sobre la cita asignada.
- *Partes de Incapacidad Temporal (IT)*. Son gestionados íntegramente por el médico desde la consulta, con la posibilidad de colaborar con el referente administrativo para garantizar eficiencia y precisión en el trámite.

Esta estructura optimiza los procesos asistenciales al completar cada gestión en un solo paso, reduciendo las listas de espera invisibles y minimizando los desplazamientos innecesarios del paciente. Asimismo, permite un mejor aprovechamiento del tiempo clínico, fortaleciendo la confianza en un equipo resolutivo y bien coordinado que actúa con agilidad y eficacia.

Línea Estratégica 4: Mejorar la accesibilidad con redefinición de las agendas profesionales.

La reorganización de las agendas asistenciales se erige como aspecto clave para la implementación efectiva del MTA. Este cambio transforma una distribución tradicional, rígida y poco adaptada a las necesidades reales, y la renueva en un sistema más segmentado, altamente dinámico y más ajustado a las particularidades de cada centro. Así, se logra ofrecer una respuesta más eficiente a las demandas de los usuarios, al tiempo que se evita la sobrecarga de los profesionales.

Como parte de la implementación del modelo asistencial, las agendas contemplan los siguientes tipos de citas, organizadas por tramos horarios y disponibilidad:

1. Citas clínicas presenciales: Consulta médica tradicional en consulta.
2. Citas telefónicas: Seguimiento o valoración médica no presencial.
3. Citas de demanda administrativa: Trámites como renovación de medicación.
4. Consulta Programada gestionada por el médico: Cita fija destinada a revisiones o seguimientos clínicos.
5. Visita Domiciliaria Programada: Atención en el domicilio para pacientes inmovilizados o crónicos.
6. Consultas no demorables (CND), disponibles durante toda la jornada (8:00-20:00 h).
7. Tramos flexibles de uso médico exclusivo, citas a discreción del facultativo.
8. Aviso a Demanda: Atención domiciliaria no programada a petición del usuario.
9. Intervención Avanzada Individual Tabaco: Cita programada por el facultativo.

Tabla S1. Composición de las Agendas Médicas en los Centros de Salud Jerez Sur, La Milagrosa y Jerez Centro de la Zona Básica Salud Jerez tras la implementación del Modelo Trinomio Asistencial (mayo 2024)


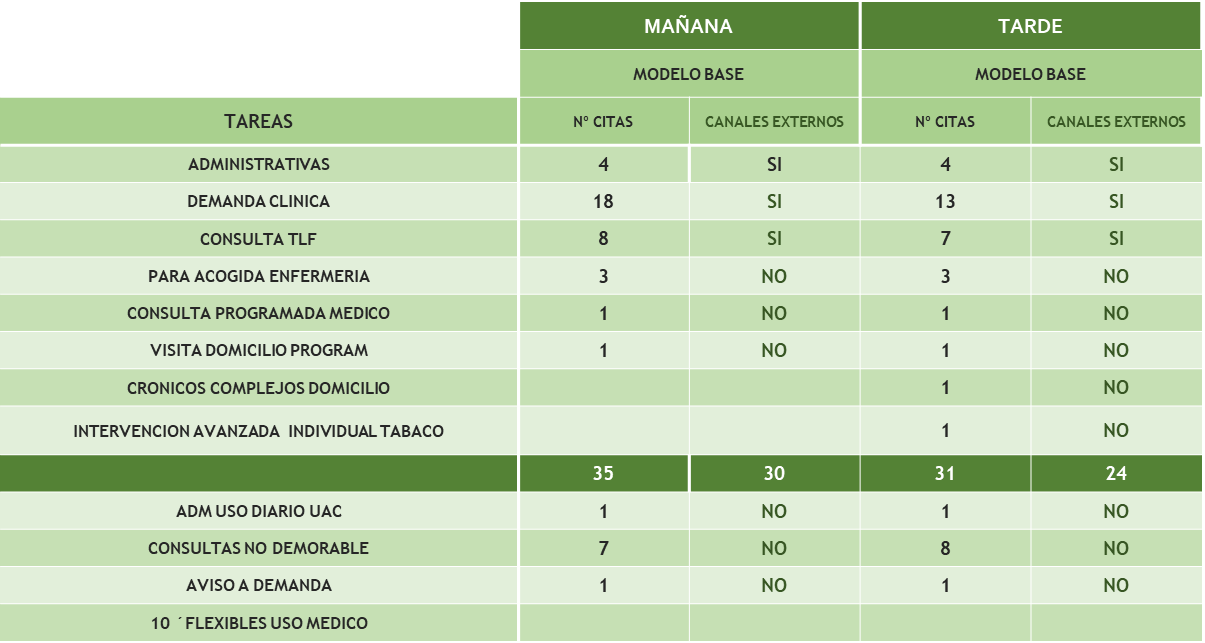


Fuente: Elaboración propia

Este diseño de agenda ajusta la disponibilidad del profesional a la demanda, anticipando huecos para casos espontáneos y facilitando la coordinación entre los tres perfiles. Además, garantiza una distribución equitativa de las consultas no demorables sin sobrecargar las agendas programadas, respetando el límite de 35 pacientes por día por facultativo según la Instrucción 0021/23 del SAS.

Línea Estratégica 5. Participación ciudadana activa, fomentando la comunicación bidireccional y el compromiso de los usuarios con el modelo

El modelo enfatiza la relevancia de la implicación activa de los usuarios en la organización y provisión de servicios asistenciales. La participación ciudadana implica que las personas usuarias asuman un rol colaborativo en los procesos asistenciales y en la mejora continua del sistema sanitario. En términos prácticos, esta participación se concreta en diversas acciones:

*Educación sanitaria y corresponsabilidad:* Durante la consulta inicial y en las subsecuentes interacciones, se proporciona información y orientación a los pacientes para fomentar el autocuidado y la toma de decisiones fundamentadas acerca de su salud. Por ejemplo, el personal de enfermería no solo se ocupa de gestionar o derivar casos, sino que también ofrece recomendaciones de autocuidado y alerta temprana sobre signos clínicos. Un paciente informado participa activamente en la gestión de su condición médica, mejorando así los resultados terapéuticos y minimizando consultas evitables. Entre las actividades sugeridas se incluyen control dietético, adherencia farmacológica y el uso adecuado de los servicios disponibles.

*Consideración de las preferencias de las personas usuarias****:*** El diseño del modelo incorpora las preferencias expresadas por los pacientes en la planificación de su atención. Un ejemplo de esta práctica es la posibilidad de elegir la modalidad de consulta programada, optando entre presencial o telefónica, según sus necesidades y preferencias específicas. Adicionalmente, se fomenta el uso de herramientas digitales entre aquellos usuarios con competencias tecnológicas, permitiendo su participación activa en el proceso asistencial, como el envío de datos clínicos a través de aplicaciones o la autogestión de citas mediante la plataforma ClicSalud+. Estas opciones no solo incrementan la comodidad de las personas usuarias, sino también su nivel de satisfacción y compromiso con el sistema sanitario.

*Retroalimentación y mejora continua del servicio:* La participación ciudadana incluye también la expresión de la opinión de los pacientes respecto a la gestión sanitaria. Los centros de salud cuentan con mecanismos destinados a recoger estas opiniones, tales como encuestas de satisfacción, buzones de sugerencias y comentarios en línea, así como consejos de participación ciudadana en los distritos sanitarios. Este proceso de retroalimentación permite a los gestores realizar ajustes en el modelo asistencial conforme a las necesidades reales de la población. Asimismo, si se identifica confusión sobre el uso de una aplicación, se refuerza la comunicación y formación para su correcta utilización. En síntesis, los pacientes contribuyen activamente en el diseño y evaluación del servicio, fortaleciendo la relación entre la comunidad y el sistema sanitario.

En el marco del MTA, y con el objetivo de garantizar una implementación óptima y fomentar la participación ciudadana efectiva, se ha indicado a las unidades que deben visualizar y comunicar el nuevo modelo a sus usuarios a través de las reuniones de participación ciudadana. Se recomienda la realización de tres reuniones anuales: la primera destinada a la presentación del modelo organizativo, y las siguientes enfocadas en obtener retroalimentación por parte de los usuarios, conocer su opinión sobre la experiencia asistencial y establecer mejoras según su visión. Esta dinámica no solo favorece una mayor implicación de la ciudadanía, sino que también convierte estas reuniones en espacios clave para el diálogo, la transparencia y la cogestión del sistema.

Adicionalmente, desde el Área de Gestión Sanitaria, se ha impulsado la visualización del modelo mediante reuniones celebradas en el hospital, en las que se ha invitado a representantes de todas las asociaciones vecinales (AAVV**)** bajo nuestra influencia territorial. Estas acciones han fortalecido el vínculo con la comunidad y han permitido recoger impresiones directas, asegurando que el modelo se alinee con las expectativas y necesidades reales de la población.

*La integración activa de los ciudadanos fomenta un sentido de cogestión sanitaria*: el sistema proporciona las herramientas y recursos necesarios, mientras que los usuarios informados los utilizan de manera adecuada y retroalimentan el sistema. Como resultado, se obtiene una atención personalizada y eficiente, junto con personas usuarias satisfechas y corresponsables del funcionamiento del modelo asistencial.

Línea Estratégica 6. Digitalización asistencial

La sexta línea estratégica del modelo responde a una necesidad creciente: adaptar la Atención Primaria a la realidad digital actual, favoreciendo que los usuarios puedan gestionar de forma más autónoma y eficiente aspectos básicos de su salud.

Hasta ahora, la relación del paciente con el sistema sanitario seguía dependiendo en gran medida del contacto presencial para trámites y consultas sencillas. Esto generaba una gran sobrecarga administrativa, desplazamientos innecesarios y tiempos de espera evitables.
La incorporación intensiva de las herramientas digitales de salud permite deslocalizar gestiones, mejorar la accesibilidad y optimizar los circuitos asistenciales. No se trata simplemente de ofrecer tecnología: el objetivo es lograr que el ciudadano sea protagonista activo de su cuidado, apropiándose de estas herramientas y utilizándolas de forma habitual y segura.

Esta línea estratégica pivota sobre la promoción intensiva del uso de tres herramientas clave:

- *ClicSalud+*: permite al paciente consultar su historia clínica electrónica, resultados de pruebas, medicación activa, gestionar citas y comunicarse mediante mensajería segura o teleconsulta.
- *App Salud Andalucía*: ofrece estas funcionalidades en el móvil, facilitando el acceso en cualquier momento.
- *Salud Responde*: servicio telefónico que complementa la atención digital para usuarios con menos competencia tecnológica.

Estas plataformas se convierten en la oficina virtual de salud del paciente, permitiéndole resolver trámites y consultas de manera autónoma, sin necesidad de acudir físicamente al centro.

Para garantizar el éxito de esta línea, el modelo integra varias acciones:

- Formación personalizada desde la UAC: Se asiste a los pacientes que presentan dificultades, facilitándoles el acceso y el uso efectivo de las herramientas digitales.
- Campañas internas de sensibilización: Se informa a los usuarios de las ventajas y utilidades prácticas de estas plataformas, animándolos a incorporarlas a su vida diaria.
- Seguimiento de uso: Se audita la utilización real de las plataformas por parte de los usuarios, detectando barreras y áreas de mejora.

La coordinación entre UAC, enfermería y medicina de familia es fundamental para consolidar este cambio, integrando la atención presencial y digital de manera fluida.

**Anexo 2. Funcionamiento operativo del modelo**

El funcionamiento operativo del Modelo Trinomio Asistencial se basa en la reorganización del circuito asistencial desde la llegada del paciente al centro de salud.

Una vez que un paciente se presenta físicamente en el centro de salud sin haber concertado una cita previa, el primer punto de contacto es el sistema de turnos, ubicado estratégicamente en la entrada del establecimiento. Por medio de este dispositivo, el usuario tiene la posibilidad de seleccionar la opción que mejor se ajuste a su requerimiento entre las siguientes alternativas (Figura 1):

- Solicitud de consulta no demorable, conocida anteriormente como "urgencias", aunque no necesariamente implique situaciones de carácter urgente.
- Gestiones administrativas, tales como trámites burocráticos o la entrega y recepción de documentación.
- Solicitud de cita programada para ser atendido en una ocasión posterior.


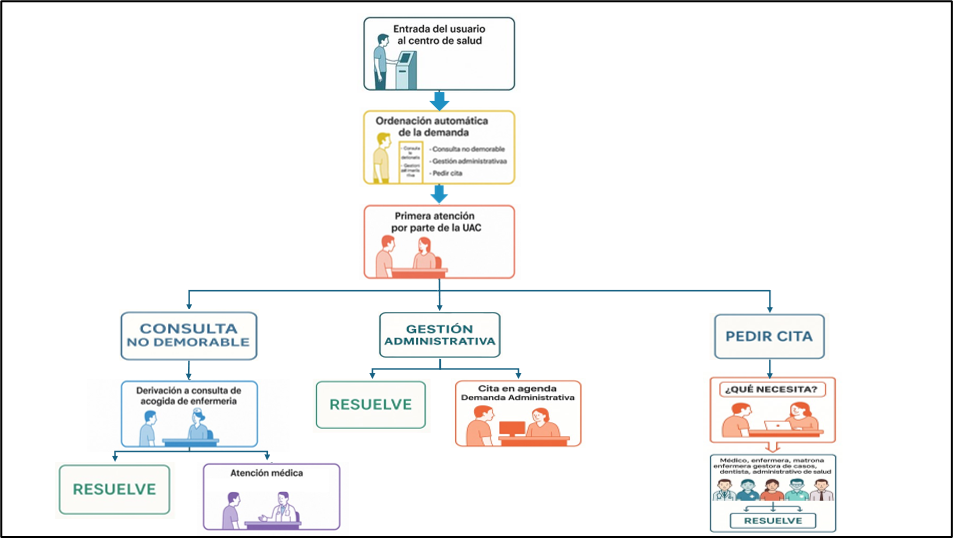


Figura S1. Flujo Operativo del Paciente tras la implementación Modelo Trinomio Asistencial (mayo 2024), en los Centros de Salud Jerez Centro, La Milagrosa y Jerez Sur de la Zona Básica Salud Jerez.

Figura 2. Flujo Operativo del Paciente

Su implementación permite evitar aglomeraciones innecesarias, optimizar los tiempos de espera y garantizar una respuesta eficiente por parte del equipo asistencial. Este mecanismo constituye, en esencia, el punto de partida del circuito asistencial establecido en el modelo.

**Anexo 3. Circuitos asistenciales**

*Circuito 1.*

Cuando el paciente selecciona la opción de "consulta no demorable", es dirigido a una mesa diferenciada atendida por un profesional de la UAC, quien realiza una primera valoración y actúa como punto de cribado y canalización asistencial. Desde este puesto:

- Se verifica la naturaleza real de la demanda expresada por el paciente.
- Se deriva directamente a la consulta de acogida de enfermería, si procede.
- Se resuelven o reconducen gestiones administrativas mal dirigidas o mezcladas con demandas clínicas.

Este contacto inicial, correctamente estructurado, evita saturaciones, reduce tiempos de espera innecesarios y filtra eficazmente la demanda.

Una vez que el paciente accede a la consulta de acogida, es atendido por un profesional de enfermería, que realiza una valoración clínica inmediata y toma decisiones según el nivel de complejidad y la naturaleza de la demanda.

Desde esta consulta, el profesional de enfermería puede adoptar varias vías de actuación:

- Si la demanda clínica puede ser resuelta desde enfermería, el proceso se da por finalizado en ese mismo acto, aportando una respuesta eficaz y sin necesidad de derivación.
- Si la demanda requiere atención médica por su propio médico, pero es demorable, el profesional de enfermería puede citar directamente al paciente en la agenda de su médico de familia ese mismo día, ya que las agendas del nuevo modelo están diseñadas para admitir este tipo de interconsultas internas y no programadas, favoreciendo la resolución en jornada.
- En caso de que, por la naturaleza de la demanda clínica, pueda ser atendido por otro médico aunque no sea su referente, el profesional de enfermería contacta de nuevo con el referente de la UAC que gestiona las consultas no demorables. A través de este canal, solicita una cita en la agenda específica de consultas no demorables médicas, diseñada para este tipo de derivaciones internas.

El profesional de la UAC, que tiene asignado el rol de gestor de estas agendas, asigna la cita de forma equitativa entre todos los médicos del equipo, garantizando así una distribución justa de la carga asistencial y evitando cuellos de botella.

Por último, el profesional de enfermería comunica al paciente la hora exacta y el número de consulta en el que será atendido, cerrando el circuito de forma organizada y fluida.

*Circuito 2.*

Cuando el paciente selecciona en el turnómetro la opción de gestión administrativa, es atendido por un profesional de la UAC, que evalúa de forma inmediata la naturaleza del trámite solicitado.

Existen dos posibles vías de actuación:

- Si el trámite puede resolverse en el momento (por ejemplo, entrega de documentación, impresión de justificantes, resolución de dudas frecuentes), el profesional de la UAC atiende directamente la demanda, ofreciendo una respuesta ágil y cerrando el proceso sin necesidad de nuevas citas.
- Si el trámite requiere una gestión más compleja o documentación que el paciente no lleva en ese momento, se le asigna una cita específica en la agenda de demanda administrativa, diseñada y gestionada por la propia UAC. En ese momento, el profesional informa al usuario del día, hora y lugar en el que será atendido, además de detallar qué documentación debe aportar para garantizar una resolución eficaz.

Este procedimiento reduce la presión sobre las consultas médicas y de enfermería, evita la derivación innecesaria de tareas administrativas al personal clínico y mejora la experiencia del usuario, al ofrecerle una atención clara, ordenada y orientada a la resolución.

*Circuito 3.*

Cuando el usuario selecciona en el turnómetro la opción **“**pedir cita”, el profesional de la UAC que lo atiende no se limita a otorgar una cita aleatoria, sino que formula una pregunta clave:
“¿Qué necesita?”

A partir de esta información, posiciona activamente la demanda en el profesional más adecuado en función de su naturaleza, dentro del equipo multidisciplinar disponible. Así, la UAC puede derivar directamente al usuario hacia:

- Médico de familia
- Enfermero de familia
- Matrona
- Trabajador social
- Odontólogo
- Administrativo de salud

Este enfoque evita errores en la asignación de citas y permite canalizar desde el inicio las necesidades hacia el profesional competente, respetando el nivel de complejidad, competencias y carga asistencial. En el caso de que el usuario manifieste una necesidad no clínica asistencial relacionada con su médico o profesional de enfermería de referencia (por ejemplo, dudas de recetas, informes, aclaraciones o dudas sobre bajas IT), el profesional de la UAC activa el circuito interno del trinomio. Es decir, contacta directamente con el referente UAC del cupo, quien actúa en coordinación con el médico y profesional de enfermería de cabecera del paciente para dar respuesta ajustada en 24-48 horas. El compromiso adquirido es que el propio referente de la UAC informa personalmente al usuario de la respuesta.

Este circuito personalizado evita derivaciones innecesarias a consulta presencial, mejora la eficiencia organizativa y consolida el funcionamiento real del trinomio asistencial como célula operativa estable, cercana y orientada a la resolución. En situaciones excepcionales, cuando el paciente presenta síntomas muy graves o se trata de una urgencia vital, el circuito habitual se interrumpe de forma inmediata. En estos casos, el personal del centro activa el protocolo específico de atención urgente, garantizando una intervención sin demoras. En Andalucía, este modelo de actuación está implantado en la mayoría de los centros de salud, permitiendo identificar y priorizar las urgencias verdaderas desde el primer momento.

Para finalizar, es importante destacar que la comunicación constante entre el profesional médico, el profesional de enfermería y el profesional de la UAC es una de las claves del modelo. Este contacto continuo permite ajustar circuitos en tiempo real, resolver incidencias de forma más ágil y garantizar una atención verdaderamente coordinada. La sensación de equipo ampliado no solo mejora la eficiencia del sistema, sino que también impacta directamente en la experiencia del paciente y en la satisfacción de los propios profesionales.

**Anexo 4. Organización de las agendas asistenciales**

La organización de las agendas asistenciales en el MTA tiene como objetivo principal mejorar la accesibilidad y la experiencia del paciente, mediante una optimización del tiempo clínico, una distribución funcional de la actividad asistencial y una distribución equitativa de la carga de trabajo, especialmente de las consultas no demorables, entre los profesionales que coinciden en un mismo turno.

**Agendas de mañana: principios organizativos**

Las agendas de mañana se estructuran a partir de dos modelos base, que cuentan con el mismo número total de citas, pero distribuidas de forma diferente en el tiempo y por tipo de actividad asistencial. Esta diferenciación no busca aumentar la capacidad asistencial, sino modular la presencia física simultánea de pacientes y facilitar una asignación equilibrada de la demanda no demorable entre los profesionales disponibles.

**Alternancia funcional de actividades**

El principio organizativo clave del modelo es la alternancia funcional de actividades. Cuando coinciden varios profesionales médicos en un mismo tramo horario, no todos realizan de forma simultánea actividad presencial. De este modo, mientras un profesional atiende consultas clínicas presenciales, otro puede estar realizando consultas telefónicas.

Esta alternancia permite que varios profesionales trabajen en paralelo sin generar picos de afluencia de pacientes presenciales, al tiempo que favorece una distribución equitativa de las consultas no demorables, evitando que recaigan de forma sistemática sobre un único profesional.

**Distribución equilibrada de la demanda presencial y no demorable**

Las consultas presenciales programadas se escalonan a lo largo de la mañana, manteniendo un flujo continuo pero controlado de pacientes. De forma complementaria, las consultas no demorables se distribuyen de manera equitativa entre los profesionales que coinciden en el turno, asignándose en distintos tramos horarios y evitando concentraciones de carga asistencial en un solo médico.

Este enfoque refuerza la corresponsabilidad del equipo y contribuye a una gestión más justa y sostenible de la demanda asistencial.

**Tipos de actividad y tiempos estándar**

En ambos modelos de agenda se utilizan tiempos homogéneos por tipo de actividad, lo que facilita la planificación y la replicabilidad del modelo:

- Consulta clínica presencial programada: 10 minutos
- Consulta no demorable: 5 minutos
- Consulta telefónica: 5 minutos
- Demanda administrativa médica: 2 minutos
- Consultas programadas de revisión: 12 minutos
- Visita programada a domicilio: 30 minutos

**Agendas de tarde: adaptación a las características del centro**

Las agendas de tarde mantienen los mismos principios organizativos que las de mañana, aplicando la alternancia de actividades y la distribución equitativa de la demanda no demorable entre los profesionales coincidentes, con adaptación al número de profesionales y a los espacios físicos disponibles.

En centros grandes, con salas de espera amplias y menor coincidencia de profesionales en horario de tarde, puede ser suficiente un único modelo de agenda. En centros con mayor coincidencia de profesionales o con limitaciones de espacio, la alternancia de modelos permite modular el flujo de pacientes y repartir de forma equilibrada la carga asistencial. El diseño del modelo permite adaptarse tanto a turnos con uno o dos profesionales como a aquellos con mayor número de médicos coincidentes.

**Visita domiciliaria a pacientes crónicos complejos**

En las agendas de tarde se incorpora de forma estructural la visita domiciliaria a pacientes crónicos complejos, planificada como actividad específica y realizada de manera conjunta por el profesional médico y la enfermera del cupo, favoreciendo un abordaje integral y coordinado.

**Finalización de agendas ordinarias y agendas dispensarizadas**

Todas las agendas ordinarias, tanto las de mañana como las de tarde, finalizan a las 13:40 horas y a las 19:10 respectivamente. A partir de ese momento, la actividad asistencial se reorganiza en agendas dispensarizadas, destinadas a atender la demanda no demorable que no haya podido ser absorbida durante el tramo ordinario.

Estas agendas son atendidas por los profesionales del mismo turno según la organización de cada centro. En centros con más de 10 profesionales médicos, esta atención recae habitualmente en dos profesionales, lo que permite mantener una distribución equitativa de la demanda no demorable residual sin sobrecargar al conjunto del equipo.

La combinación de agendas estructuradas por tipo de actividad, alternancia funcional según el número de profesionales y los espacios disponibles, visitas domiciliarias conjuntas y agendas dispensarizadas permite una gestión integral, equitativa y sostenible de la jornada asistencial, aplicable a centros de distinto tamaño y complejidad organizativa, sin necesidad de incrementar recursos y manteniendo una mejora significativa de la accesibilidad.

**Anexo 5. Resultados cuantitativos complementarios**

A continuación se presentan los análisis gráficos complementarios de la variación del Tiempo Medio de Respuesta (TMR). Las figuras se proporcionan también como archivos independientes.


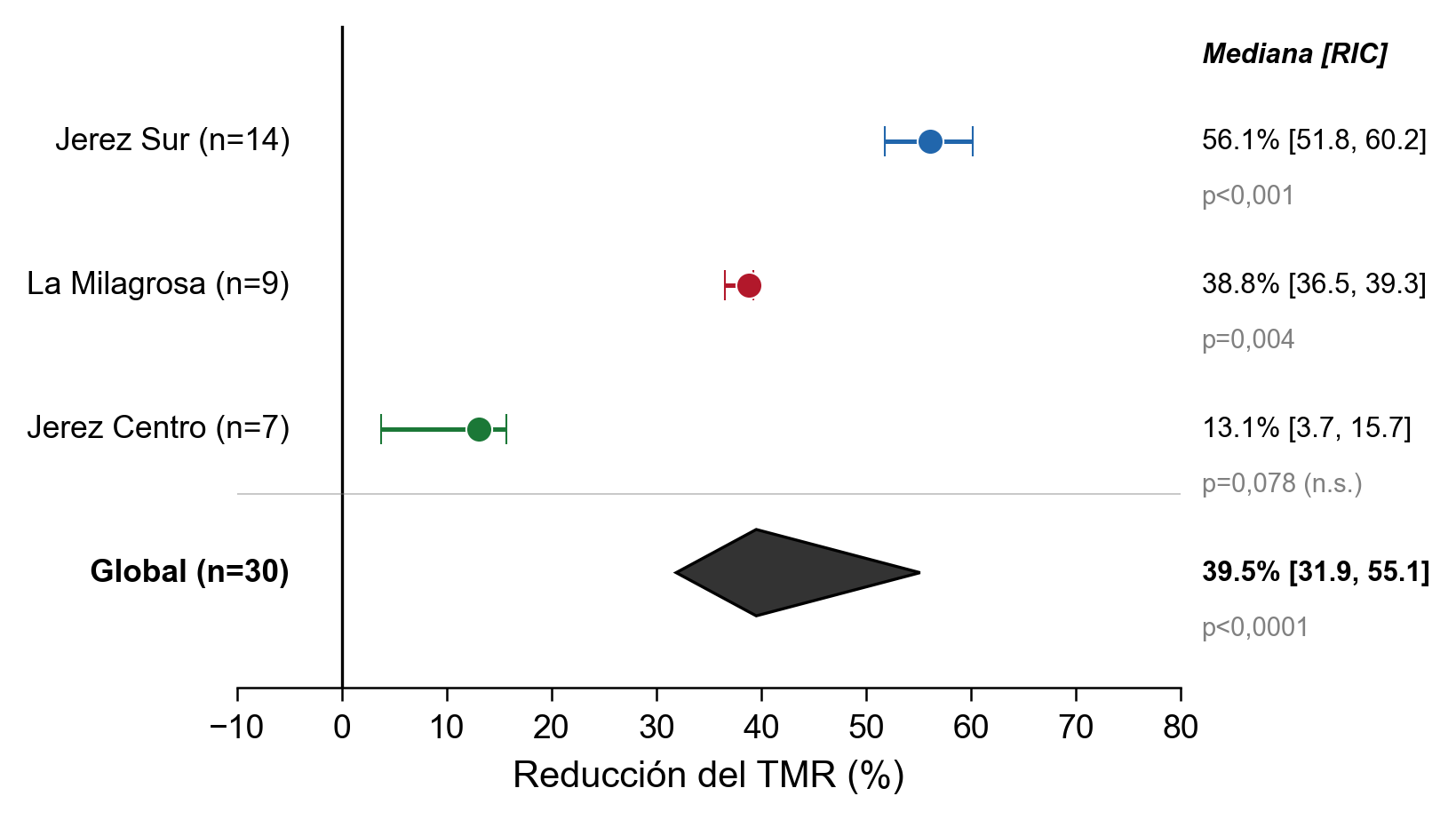


*Figura S1. Forest plot: efecto del Modelo Trinomio Asistencial sobre el TMR por centro de salud y estimación global. El diamante representa la mediana global con su rango intercuartil.*


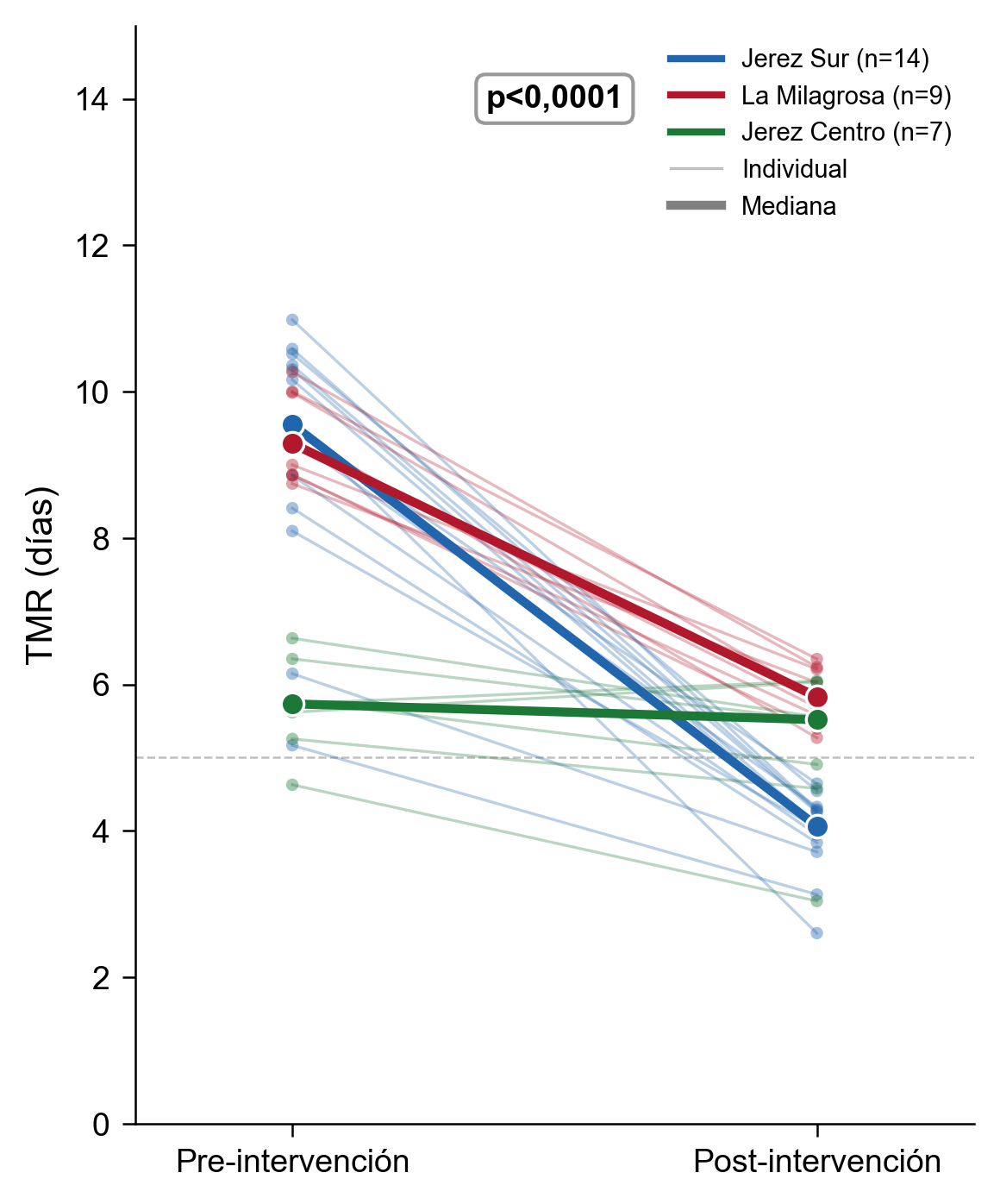


*Figura S2. Gráfico de pendientes (spaghetti): evolución individual del TMR antes y después de la intervención para los 30 profesionales incluidos, diferenciados por centro de salud. Las líneas gruesas representan las medianas por centro.*


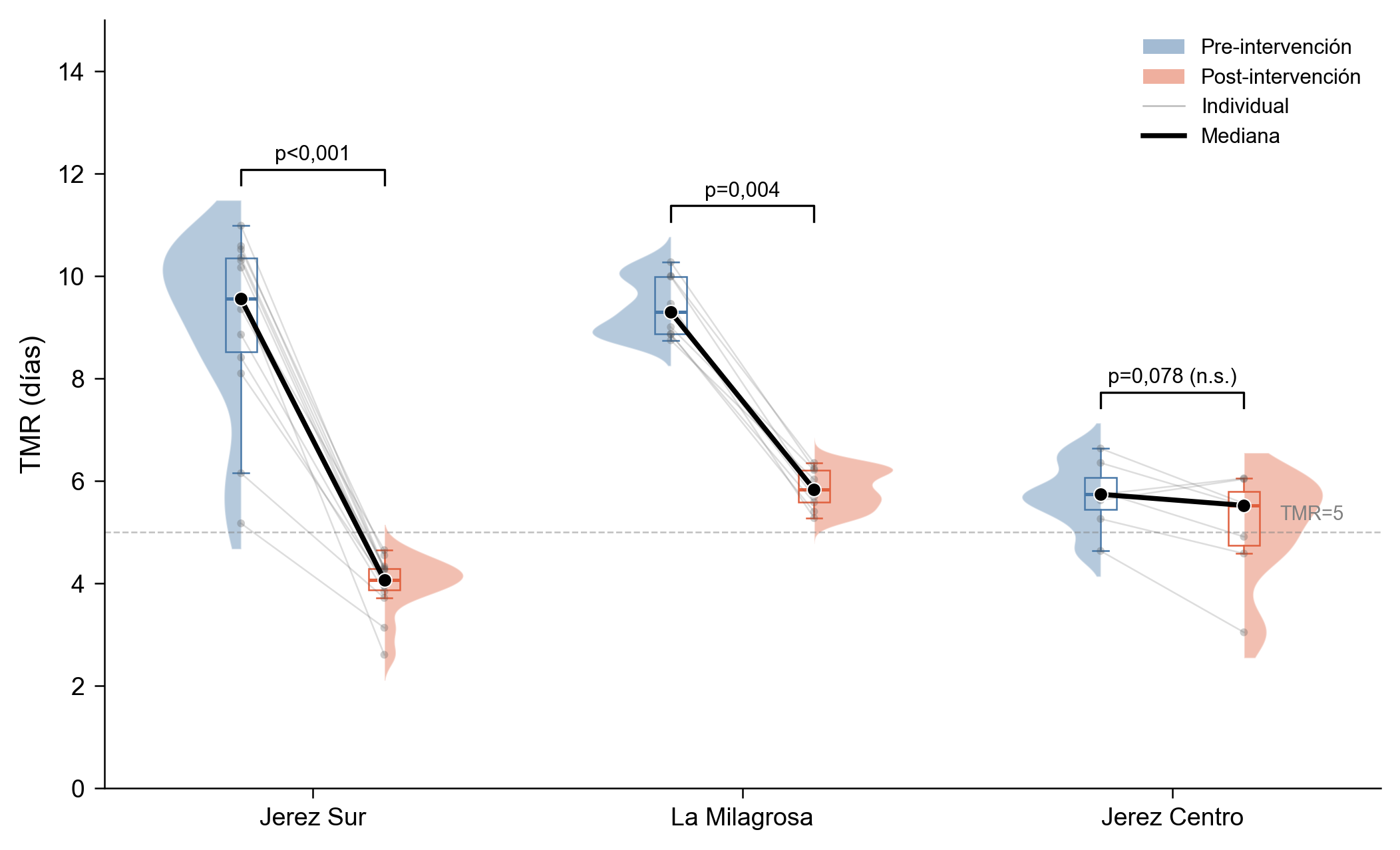


*Figura S3. Diagrama combinado de violín con líneas pareadas: distribución del TMR y evolución individual antes y después de la intervención, por centro de salud. La línea horizontal discontinua indica el umbral de TMR = 5 días.*

Listado de tablas suplementarias

Tabla S1. Composición de las Agendas Médicas en los Centros de Salud Jerez Sur, La Milagrosa y Jerez Centro de la Zona Básica Salud Jerez tras la implementación del Modelo Trinomio Asistencial.

Listado de figuras suplementarias

Figura A1. Flujo operativo del paciente tras la implementación del Modelo Trinomio Asistencial en los centros de salud de la Zona Básica de Salud de Jerez.

Figura S1. Forest plot: efecto del Modelo Trinomio Asistencial sobre el TMR por centro de salud y estimación global.

Figura S2. Gráfico de pendientes (spaghetti): evolución individual del TMR antes y después de la intervención para los 30 profesionales incluidos, por centro de salud.

Figura S3. Diagrama combinado de violín con líneas pareadas: distribución del TMR y evolución individual antes y después de la intervención, por centro de salud.
